# Effect of Marital Status on Upper Digestive Tract Tumor Survival: Married Male Patients Exhibited a Better Prognosis

**DOI:** 10.3389/fsurg.2022.880893

**Published:** 2022-04-11

**Authors:** Maofeng Qing, Jiakuan Peng, Qianhui Shang, Hao Xu, Qianming Chen

**Affiliations:** State Key Laboratory of Oral Diseases, National Clinical Research Center for Oral Diseases, West China Hospital of Stomatology, Sichuan University, Chengdu, China

**Keywords:** upper digestive tract tumors, marital status, SEER, prognostic factors, cancer-specific survival

## Abstract

**Purpose:**

Marital status has been associated with the outcomes in several types of cancer, but less is known about upper digestive tract tumors (UDTTs). The study aims to explore the effect of marital status on the survival outcomes of UDTT.

**Methods:**

We collected patient cases of UDTT using the Surveillance, Epidemiology, and End Results (SEER) database between 1975 and 2016. The univariate analyses of overall survival (OS) and cancer-specific survival (CSS) were performed using the Kaplan–Meier method. The multivariate survival analyses were performed using Cox proportional hazard model.

**Results:**

A total of 282,189 patients were included, with 56.42, 16.30, 13.33, and 13.95% of patients married, never married, divorced or separated, and widowed, respectively. The significant differences were observed among married, never-married, divorced or separated, and widowed patients with regard to the year of diagnosis, sex, age, race, pathological type, anatomical site, the number of primary tumor, grade, rate of surgery performed, radiotherapy, chemotherapy (*p* < 0.001). The proportions of patients with 3-year and 5-year OS were 54.22 and 48.02% in the married group, 46.96 and 41.12% in the never-married group, 44.24 and 38.06% in the divorced or separated group, 34.59 and 27.57% in the widowed group, respectively (*p* < 0.001); the proportions of patients with 3-year and 5-year CSS were 70.76 and 68.13% in the married group, 62.44 and 59,93% in the never-married group, 63.13 and 60.53% in the divorced or separated group, 62.11 and 58.89% in the widowed group, respectively (*p* < 0.001); all these data indicated married patients exhibited favorable OS and CSS than never-married, divorced or separated, and widowed patients. Men in the married group showed better OS (*HR*, 1.16; 95%CI: 1.11–1.22) and CSS (*HR*, 0.96; 95%CI: 0.92–1.23) than those in the never-married group.

**Conclusion:**

This study reveals that marital status is an independent prognostic factor for OS and CSS of patients with UDTT. Married male patients with UDTT trend to have a better prognosis.

## Introduction

Social supports are emerging and closely related to the cancer prognosis as the society develops attracting more attention (1, 2). Marital status as one of the most important social relationships has significant implications for human health and well-being. The numerous studies have identified significant differences in morbidity and prognosis of different diseases in different marital statuses (3–6). Aizer et al. used the Surveillance, Epidemiology, and End Results (SEER) database for analysis and found that unmarried patients have higher risks of cancer metastasis, under-treatment, and death compared to married patients in a sample size of nearly 1 million patients (7). Marital status has been increasingly considered as an independent factor in the prognostic assessment of many cancers (7–9).

The upper digestive tract tumors (UDTTs), accounted for 6.8% of new on-set cancers and 8.9% of cancer deaths worldwide in 2018, are the seventh most frequent cancer type and the seventh most common cause of death from cancer worldwide (10). The upper digestive tract (UDT), which includes oral cavity, larynx, and esophagus, is the passage through which food enters the body and is covered by squamous epithelium, and the most frequent pathological type of UDTT is squamous cell carcinoma (SCC) (11–13). Sociological behaviors and psychosocial factors such as smoking, drinking, HPV infection, and upset emotion contribute enormously to UDTT (14–18). Psychosocial factors are involved in the pathogenesis of mental disorders by acting *via* mechanisms involving epigenetics (19), and the impact of different marital statuses on sociological behavior and psychosocial factor is critical (20, 21). Marriage has been a protective factor in many previous studies of cancer associations (7, 8). However, as far as we are concerned, first, UDTT is closely related to many sociological factors, and whether marriage, as an important sociological factor, is related to UDTT has not been studied before. Second, on the research methods, many other tumor-related studies only discuss the relationship between marital status and overall survival (OS) rate, but the lack of research on relationship about marital status and cancer-specific survival (CSS) rate which is better at showing the relationship between the tumor and survival. Therefore, the aim of our study was to explore the effects of different marital statuses, which include married, never married, divorced or separated, widowed on the prognosis of patients with UDTTs according to the multiple stratified studies based on the SEER population-based database.

## Materials and Methods

### Patient Selection and Data Collection

**Figure 1:**
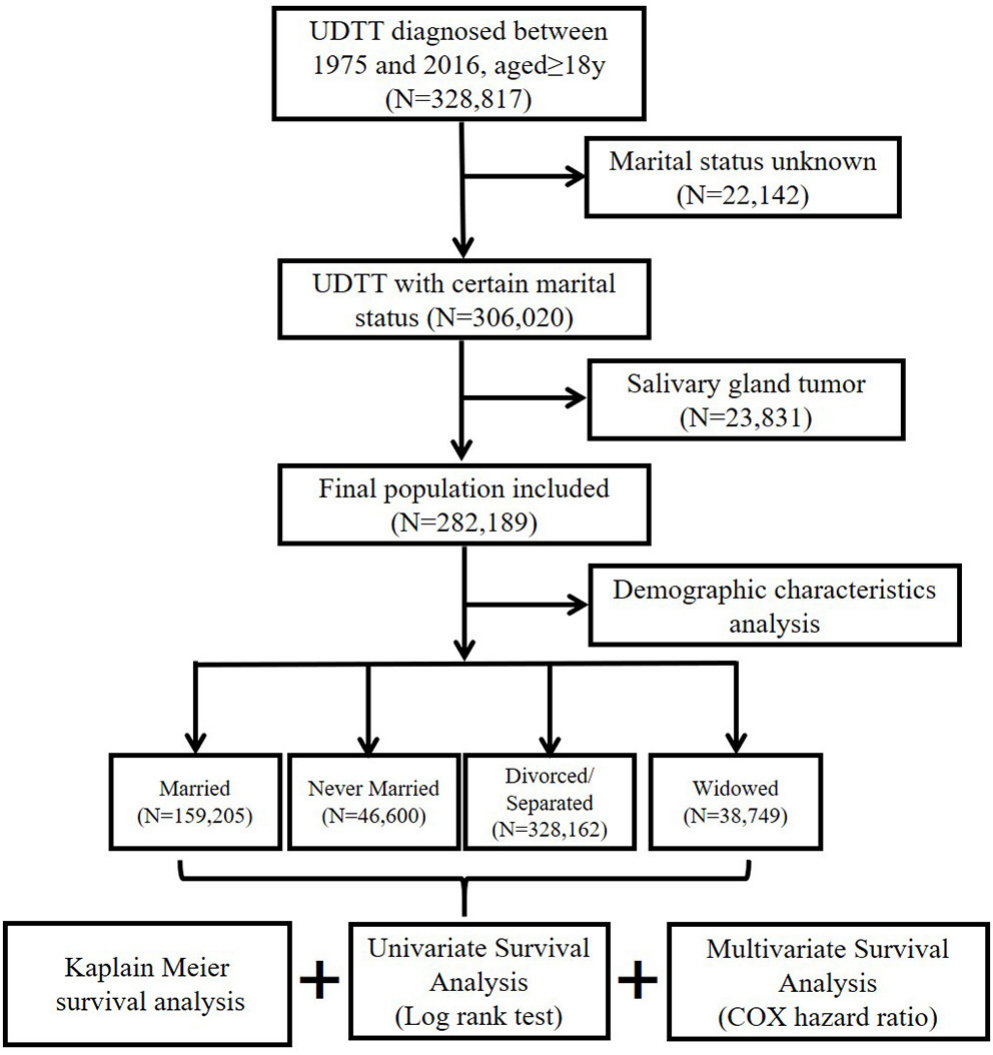
Eligibility criteria for collection of patients with UDTTs.

Patient cases of UDTT included between 1975 and 2016 were collected from the SEER database, which is the largest population-based cancer registry in the world and included 18 cancer registries as released on 2019 (22). UDT is comprised of three anatomical sites: (1) oral department consisting of lip (C000–C009), tongue (C019–C029), and floor of mouth (C040–C049); (2) pharyngeal department consisting of nasopharynx (C110–C119), tonsil (C090–C099), oropharynx (C100–C109), hypopharynx (C129–C130–139), and other oral cavity and pharynx (C140, C142–C148); (3) esophagus (C150–C159). Accordingly, three groups were classified based on the anatomical sites, oral, pharyngeal, and esophagus. Cases with different histological types were identified using the codes of International Classification of Oncology (ICD-O-3/WHO 2008) for tumor morphology (SCC = 8050–8084). Tumors of the salivary gland were excluded due to the differences in the type of epithelium, pathological type, and etiology. The flow chart is shown in Figure 1. Collected UDTT patients must have age more than 18 years, with demographic and clinical information that includes years of diagnosis, age, sex, race, marital status, anatomical site, pathological type, number of primary tumors, grade, stage, surgery, radiotherapy, chemotherapy, 3-year OS rate, 5-year OS, 3-year CSS rate, 5-year CSS, total OS rate, and total CSS rate. Grade was rated from I to IV based on the cancer cell differentiation. Stage was also rated from I to IV based on tumor metastasis. OS or CSS was defined as the date when the patient diagnosed with cancer to the date of the patient's death or cancer-specific death. When CSS calculated, deaths from other causes were treated as censored observations. It should be pointed out that, in many studies of UDTT and cancers, 45 and 75 are respectively regarded as the recognized age of a young patient and old patient (10, 23). Patients were age-stratified into <45 years, 45–60 years, 61–75 years, and >75 years. Marital status was classified as married, never married including those reported to cohabitate with an unmarried, domestic partner (same gender, opposite gender, or unregistered), divorced or separated, and widowed. Patients should be excluded if they had unknown parameters of stage, partnership status, treatment, or performance of sentinel lymph node biopsy.

### Statistical Analysis

Categorical data are shown as percentage and analyzed by chi-square test. The OS and CSS of patients were plotted using the Kaplan–Meier method according to the different marital statuses and examined by log rank test. The survival is calculated as the number of months after the date of cancer diagnosis until the date of death in the SEER. Multivariate analysis was conducted to calculate OS rate and CSS rate with Cox proportional hazard ratios (HRs), respectively, and the nomograms were plotted to show the results of Cox regression. All statistical analyses were performed with R version 3.6.8 (R Core Team, Vienna, Austria), and the significance level was set as 0.05.

## Results

### Patient Characteristics

A total of 282,189 eligible patients with UDTTs in the SEER database during a 42-year study period (1975–2016) were analyzed in this study. Their demographic and clinical information for all study population is shown in Table 1. The proportions were 56.42% (159,205/282,189) for married patients, 16.51% (46,600/282,189) for never-married patients, 13.33% (37,635/282,189) for divorced or separated patients, and 13.74% (38,749/282,189) for widowed patients. The highest proportion of new diagnoses of UDTTs was found in the period of 2002–2016 (58.52%, 165,131/282,189), possibly due to the advances in diagnose of UDTTs. In each diagnosis period with a 14-year interval, married patients accounted for the majority (*p* <0.001). The proportion of never-married patients continued to increase, from 11.31% at the beginning to 18.48% at the end. The male patients accounted for 72.13% (203,557/282,189). With regard to age stratification, new diagnoses of UDTTs were found in age ranging from 45 to 75 years (73.92% in total). There were 82.57% of patients (232,998/282189) being white, 75.75% (213,746/282189) with SCC, 42.84% (120,901/282189) occurring in oral cavity, 69.22% (195,341/282189) with a primary lesion, 33.89% (95,637/282189) with grade II, and 19.63% (55,400/282189) with stage IV (*p* < 0.001).

**Table 1:** Demographic characteristics for patient cases with UDTT by marital status.

**Characteristic**	**Total**	**Married**	**Never married**	**Divorced/Separated**	**Widowed**	***P*** **value**
	***n*** **= 282,189**	***n*** **= 159,205 (56.42%)**	***n*** **= 46,600 (16.51%)**	***n*** **= 37,635 (13.33%)**	***n*** **= 38,749 (13.74%)**	
Years of diagnosis						
1975–1988	41,164 (14.59%)	24,064 (58.46%)	4,654 (11.31%)	5,453 (13.25%)	6,993 (16.98%)	<0.001
1989–2002	75,894 (26.89%)	42,472 (55.96%)	11,437 (15.07%)	9,645 (12.71%)	12,340 (16.26%)	
2003–2016	165,131 (58.52%)	92,669 (56.12%)	30,509 (18.48%)	22,537 (13.65%)	19,416 (11.75%)	
Sex						<0.001
Male	203,557 (72.13%)	125,706 (61.75%)	35,444 (17.41%)	27,162 (13.34%)	15,245 (7.5%)	
Female	78,632 (27.87%)	33,499 (42.60%)	11,156 (14.19)	10,473 (13.32%)	23,504 (29.89%)	
Age						<0.001
<45	16,736 (5.93%)	9,042 (54.03%)	5,828 (34.82%)	1,747 (10.44%)	119 (0.78%)	
45–60	94,113 (33.35%)	53,879 (57.25%)	20,986 (22.30%)	15,738 (16.72%)	3,510 (3.73%)	
61–75	114,489 (40.57%)	68,991 (60.30%)	15,053 (13.15%)	16,009 (13.98%)	14,436 (12.57%)	
>75	56,851 (20.15%)	27,293 (48.01%)	4,733 (8.33%)	4,141 (7.28%)	20,684 (36.38%)	
Race						<0.001
White	232,998 (82.57%)	136,386 (58.53%)	33,775 (14.50%)	30,494 (13.09%)	32,343 (13.88%)	
Black	30,466 (10.80%)	10,269 (33.71%)	10,172 (33.39%)	5,723 (18.78%)	4,302 (14.12%)	
Other^*^	18,725 (6.63%)	12,550 (7.88%)	2,653 (5.69%)	1,418 (3.76%)	2,104 (5.43%)	
Pathologic type						<0.001
SCC	213,746 (75.75%)	117,851 (55.14%)	36,571 (17.11%)	30,002 (14.04%)	29,322 (13.71%)	
Non-SCC	68,443 (24.25%)	41,354 (60.42%)	10,029 (14.65%)	7,633 (11.15%)	9,427 (13.78%)	
Site						<0.001
Oral carvity	120,901 (42.84%)	68,533 (56.69%)	19,433 (16.07%)	15,557 (12.87%)	17,378 (14.37%)	
Pharynx	74,844 (26.52%)	41,838 (55.90%)	13,980 (18.68%)	11,368 (15.19%)	7,558 (10.23%)	
Esophagus	86,444 (30.64%)	48,834 (56.50%)	13,187 (15.25%)	10,710 (12.40%)	13,713 (15.85%)	
Primary number						<0.001
1	195,341 (69.22%)	108,368 (55.48%)	34,921 (17.88%)	26,450 (13.54%)	25,602 (13.10%)	
≥2	86,848 (30.78%)	50,837 (58.54%)	11,679 (13.45%)	11,185 (12.88%)	13,147 (15.14%)	
Grade						<0.001
I	31,674 (11.22%)	18,515 (58.45%)	4,527 (14.29%)	3,619 (11.43%)	5,013 (15.83%)	
II	95,637 (33.89%)	52,512 (54.91%)	16,693 (17.45%)	13,475 (14.09%)	12,957 (13.55%)	
III	80,760 (28.62%)	47,166 (58.40%)	13,111 (16.23%)	10,739 (13.30%)	9,711 (12.07%)	
IV	7,053 (2.50%)	4,397 (62.34%)	1,169 (16.58%)	719 (10.19%)	768 (10.89%)	
Unknown	67,065 (23.77%)	36,615 (54.60%)	11,100 (16.55%)	9,083 (13.54%)	1,0267 (15.31%)	
Stage						<0.001
I	23,879 (8.46%)	14,358 (60.13%)	3,688 (15.44%)	2,681 (11.23%)	3,152 (13.20%)	
II	16,968 (6.01%)	9,793 (57.71%)	2,785 (16.41%)	2,160 (12.73%)	2,230 (13.15%)	
III	22,329 (7.91%)	12,962 (58.05%)	4,008 (17.95%)	3,065 (13.73%)	2,294 (10.27%)	
IV	55,400 (19.63%)	30,378 (54.83%)	11,452 (20.67%)	8,498 (15.33%)	5,072 (9.17%)	
Unknown	163,613 (57.98%)	91,714 (56.06%)	24,667 (15.07%)	21,231 (12.98%)	26,001 (15.89%)	
Surgery						<0.001
Not performed	152,557 (54.05%)	79,617 (52.19%)	27,622 (18.11%)	21,957 (14.39%)	23,361 (15.31%)	
Performed	129,632 (45.95%)	79,588 (61.40%)	18,978 (14.64%)	15,678 (12.09%)	15,388 (11.87%)	
Radiotherapy						<0.001
No/Unknown	122,149 (43.29%)	67,030 (54.88%)	20,161 (16.51%)	14,851 (12.16%)	20,107 (16.46%)	
Yes	160,040 (56.71%)	92,175 (57.59%)	26,439 (16.52%)	22,784 (14.24%)	18,642 (11.65%)	
Chemotherapy						<0.001
No/Unknown	171,855 (60.90%)	94,041 (54.72%)	26,925 (15.67%)	21,850 (12.71%)	29,039 (16.90%)	
Yes	110,334 (39.10%)	65,164 (59.06%)	19,675 (17.83%)	15,785 (14.31%)	9,710 (8.80%)	

* *Including other (American Indian/AK Native, Asian/Pacific Islander) and unknowns*.

### Association Between Patient's Characteristics and Marital Status

Significant differences were observed among married, never-married, divorced or separated, and widowed patients with regard to the year of diagnosis, sex, age, race, pathological type, anatomical site, number of primary tumor, grade, rate of surgery performed, radiotherapy, and chemotherapy (Table 1, *p* < 0.001). In detail, a higher proportion of married patients and a lower proportion of never-married patients were found in the diagnosis period of 1975–1988 compared with other two periods (*p* < 0.001). A higher proportion of never-married patients and a lower proportion of widowed patients were found in the diagnosis period of 2003–2016 compared with other two periods (*p* < 0.001). Men had higher proportions in married (61.75 vs. 42.60%, *p* < 0.001) and never-married (17.41 vs. 14.19%, *p* < 0.001) patients but a lower proportion in widowed patients (7.5 vs. 29.89%, *p* < 0.001) when comparable to women. In each marital status, four age stratifications showed significant differences, so did race (*p* < 0.001). Higher proportions of SCC cases were noted in never-married (17.11 vs. 14.65%, *p* < 0.001) and divorced or separated (14.04 vs. 11.15%, *p* < 0.001) patients but a lower proportion of SCC cases were noted in married patients (55.14 vs. 60.42%, *p* < 0.001) than non-SCC cases. Most patients were diagnosed with grade IV in married group (62.34%), fewest patients with grade II in never-married group (14.29%), and fewest patients with grade IV in divorced or separated group (10.19%) and widowed group (10.89%). Concerning tumor stage, most stage I cases were found in married group (60.13%), most stage IV cases in never-married group (20.67%), and fewest stage IV cases in widowed group (9.17%). The proportion of patients undergoing surgery was higher than those not in married group (61.40 vs. 52.19%, *p* < 0.001), while the proportion of patients undergoing surgery was lower than those not in never-married (18.11 vs. 14.64%, *p* < 0.001), divorced or separated (14.39 vs. 12.09%, *p* < 0.001), and widowed (15.31 vs. 11.87%, *p* < 0.001) patients. More patients underwent radiotherapy and chemotherapy in married group (59.06 vs. 54.72%, *p* < 0.001), but fewer in widowed group (8.80 vs. 16.90%, *p* < 0.001).

### Association Between Patient's Characteristics, Marital Status, and Survival Outcomes of Patients With UDTTs

**Figure 2:**
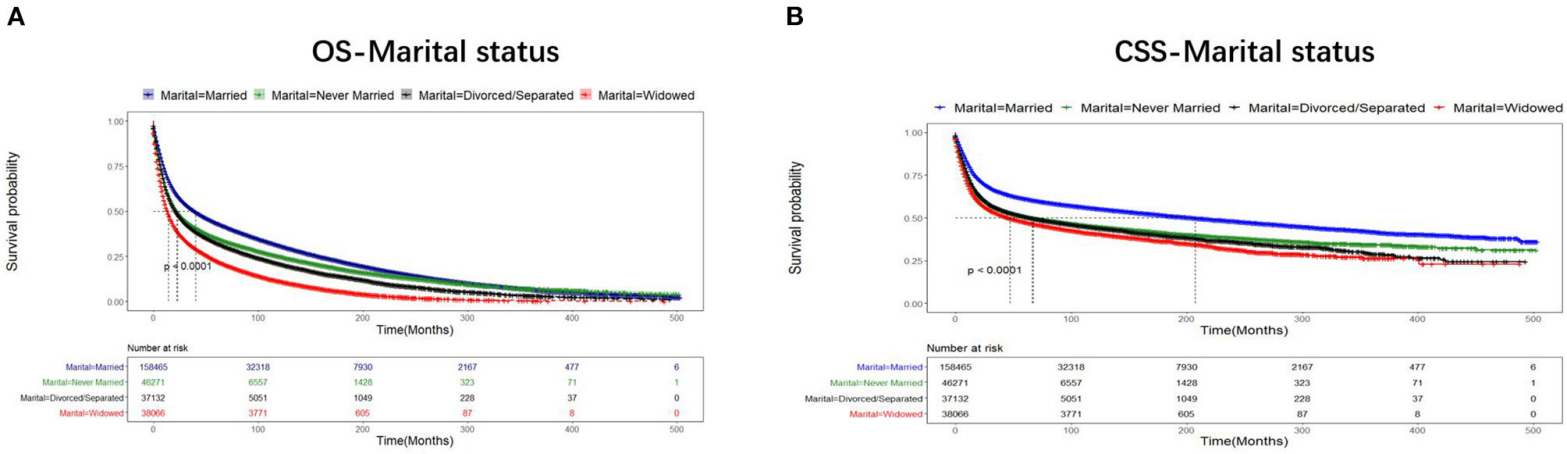
Comparison of OS and CSS of the patients with UDTT in different marital statuses, 1975–2016 (*p* < 0.001). Censoring marks are indicated with small vertical lines. **(A)** OS- Marital status; **(B)** CSS- Marital status.

The median survival time of all patients with UDTTs was 21 months, the median survival time was 26 months in the married group, 17 months in the never-married group, 17 months in the divorced or separated group, and 12 months in widowed group. As shown in Figure 2, the proportions of patients with 3-year and 5-year OS were 54.22 and 48.02% in the married group, 46.96 and 41.12% in the never-married group, 44.24 and 38.06% in the divorced or separated group, 34.59 and 27.57% in the widowed group, respectively (*p* < 0.001), which indicated that married patients exhibited favorable OS than never-married, divorced or separated, and widowed patients. Likewise, the proportions of patients with 3-year and 5-year CSS were 70.76 and 68.13% in the married group, 62.44 and 59.93% in the never-married group, 63.13 and 60.53% in the divorced or separated group, 62.11 and 58.89% in the widowed group, respectively (*p* < 0.001), which indicated that married patients exhibited favorable CSS than never-married, divorced or separated, and widowed patients. An early year of diagnosis (1975–1988), male, age older than 75 years, Black race, widowed, non-SCC, tumor in esophagus, number of primary tumors=1, tumor grade=III, stage IV, surgery not performed, and radiotherapy were considered as the significant risk factors (*p* < 0.001) of OS and CSS rate. Furthermore, chemotherapy predicted better 3-year OS rate, but worse rates of 5-year OS, 3-year CSS, and 5-year CSS (Table 2).

**Table 2:** Univariate survival analyses of patients with UDTT according to various clinicopathological variables.

**Variables**	**n**	**OS-3 years**	**OS-5 years**	**Log rank χ2 test**	***P***	**CSS- 3 years**	**CSS- 5 years**	**Log rank χ2 test**	***P***
Years of diagnosis				1808.118	<0.001			1347.515	<0.001
1975–1988	41,164	40.13%	32.27%			60.48%	57.47%		
1989–2002	75,894	43.02%	35.30%			63.29%	60.06%		
2003–2016	165,131	53.77%	48.80%			70.64%	68.52%		
Sex				116.401	<0.001			289.070	<0.001
Male	203,557	48.31%	42.18%			66.28%	63.65%		
Female	78,632	50.39%	44.26%			69.51%	67.19%		
Age				18879.218	<0.001			3304.258	<0.001
<45	16,736	69.39%	65.22%			74.61%	71.75%		
45–60	94,113	57.32%	51.88%			68.21%	65.19%		
61–75	114,489	46.98%	40.46%			66.67%	64.16%		
>75	56,851	32.76%	25.69%			63.32%	62.56%		
Race				4463.418	<0.001			4107.093	<0.001
White	232,998	50.33%	44.04%			68.74%	66.26%		
Black	30,466	33.16%	27.95%			53.88%	51.35%		
Other^*^	18,725	56.56%	50.95%			69.41%	65.99%		
Marital status				8234.502	<0.001			3963.207	<0.001
Married	159,205	54.22%	48.05%			70.76%	68.13%		
Never married	46,600	46.96%	41.12%			62.44%	59.93%		
Divorced/Sepratated	37,635	44.24%	38.06%			63.13%	60.53%		
Widowed	38,749	34.59%	27.57%			62.11%	59.89%		
Pathological type			10013.912	<0.001			12162.172	<0.001
SCC	213,746	52.98%	46.29%			71.37%	68.78%		
Non-SCC	68,443	36.11%	31.73%			54.10%	51.67%		
Site				57625.713	<0.001			54541.736	<0.001
Oral carvity	120,901	62.90%	55.54%			79.89%	77.47%		
Pharynx	74,844	56.83%	50.16%			72.94%	69.79%		
Esophagus	86,444	22.42%	18.50%			44.42%	42.21%		
Primaries number				1797.913	<0.001			35763.428	<0.001
1	195,341	46.47%	41.41%			54.86%	51.87%		
≥2	86,848	54.35%	45.81%			94.89%	94.03%		
Grade				4939.578	<0.001			5681.069	<0.001
I	31,674	64.03%	56.60%			80.27%	78.26%		
II	95,637	48.56%	42.03%			67.98%	65.29%		
III	80,760	41.65%	36.31%			59.84%	57.18%		
IV	7,053	51.30%	45.65%			65.86%	62.25%		
Unknown	67,065	50.68%	38.97%			68.85%	66.47%		
Stage				6301.868	<0.001			7478.189	<0.001
I	23,879	70.63%	64.06%			87.55%	85.77%		
II	16,968	56.02%	49.01%			75.84%	72.90%		
III	22,329	49.71%	44.35%			66.77%	64.04%		
IV	55,400	43.50%	39.54%			59.43%	57.32%		
Unknown	163,613	46.49%	39.88%			65.99%	63.25%		
Surgery				38200.422	<0.001			32165.19	<0.001
Not performed	152,557	34.54%	29.86%			57.62%	55.44%		
Performed	129,632	65.77%	57.94%			80.55%	77.65%		
Radiotherapy				211.477	<0.001			196.963	<0.001
No/Unknown	122,149	49.17%	43.40%			70.13%	68.43%		
Yes	160,040	48.68%	42.27%			64.93%	61.73%		
Chemotherapy				110.581	<0.001			1,731.93	<0.001
No/Unknown	171,855	50.34%	43.77%			70.67%	68.42%		
Yes	110,334	51.29%	41.19%			61.75%	58.73%		

* *Including other (American Indian/AK Native, Asian/Pacific Islander) and unknowns*.

### Marital Status as an Independent Factor Influencing Survival Outcomes of Patients With UDTTs

**Figure 3:**
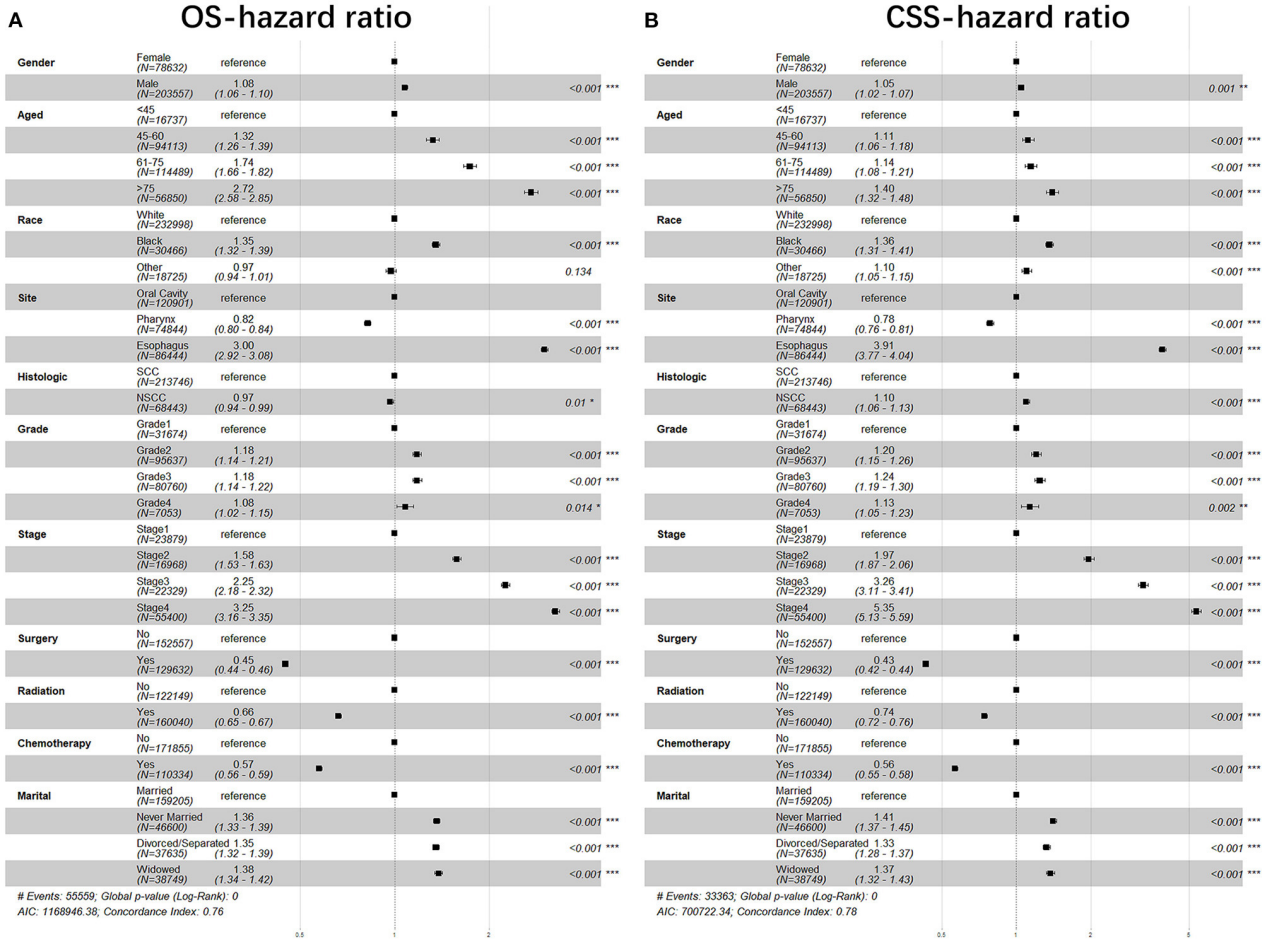
The forest plot of HR (95% CI) for OS and CSS of the patients with UDTT. HR, hazard ratio; CI, confidence interval. **(A)** OS- hazard ratio; **(B)** CSS- hazard ratio.

The variables that include sex, age, race, anatomical site, pathological type, grade, stage, surgery, radiotherapy, chemotherapy, and marital status were analyzed with Cox regression model for their correlations with the prognosis of patients with UDTTs. All variables were found to be independent prognostic factors of OS of patients with UDTTs (Figure 3, Table 3): sex (male: *HR*, 1.08; 95%CI: 1.06–1.10), age (45–60 years: *HR*, 1.32; 95%CI: 1.26–1.39; 61–75 years: *HR*, 1.74; 95%CI: 1.66–1.82; > 75 years: *HR*, 2.72; 95%CI: 2.58–2.85), race (Black: *HR*, 1.35; 95%CI: 1.32–1.39; other: *HR*, 0.97; 95%CI: 0.94–1.01), anatomical site (pharynx: *HR*, 0.82; 95%CI: 0.80–0.84; esophagus: *HR*, 3.00; 95%CI: 2.92–3.08), pathological type (NSCC: *HR*, 0.97; 95%CI: 0.94–0.99), grade (II: *HR*, 1.18; 95%CI: 1.14–1.21; III: *HR*, 1.18; 95%CI: 1.14–1.22; IV: *HR*, 1.08; 95%CI: 1.02–1.15;), stage (II: *HR*, 1.58; 95%CI: 1.53–1.63; III: *HR*, 2.25; 95%CI: 2.18–2.32; IV: *HR*, 3.25; 95%CI: 3.16–3.35;), surgery (performed: *HR*, 0.45; 95%CI: 0.44–0.46), radiotherapy (Yes: *HR*, 0.66; 95%CI: 0.65–0.67), chemotherapy (yes: *HR*, 0.57; 95%CI: 0.56–0.59), and marital status (never married: *HR*,1.36; 95%CI: 1.33–1.39; divorced/separated: *HR*, 1.35; 95%CI: 1.32–1.39; widowed: HR, 1.38; 95%CI: 1.34–1.42). Only in race, other race showed no significant difference (*p*=0.134), and other variables are significantly different. Likewise, all variables were found to be the independent prognostic factors of CSS of patients with UDTTs: sex (male: *HR*, 1.05; 95%CI: 1.02–1.07), age (45–60 years: *HR*, 1.11; 95%CI: 1.06–1.18; 61–75 years: *HR*, 1.74; 95%CI: 1.08–1.21; > 75 years: *HR*, 1.40; 95%CI: 1.32–1.48), race (Black: *HR*, 1.36; 95%CI: 1.31–1.41; other: *HR*, 1.10; 95%CI: 1.05–1.15), anatomical site (pharynx: *HR*, 0.78; 95%CI: 0.76–0.81; esophagus: *HR*, 3.91; 95%CI: 3.77–4.04), pathological type (NSCC: *HR*, 1.10; 95%CI: 1.06–1.13), grade (II: *HR*, 1.20; 95%CI: 1.15–1.26; III: *HR*, 1.24; 95%CI: 1.19–1.30; IV: *HR*, 1.13; 95%CI: 1.05–1.23), stage (II: *HR*, 1.97; 95%CI: 1.87–2.06; III: *HR*, 3.26; 95%CI: 3.11–3.41; IV: *HR*, 5.35; 95%CI: 5.13–5.59;), surgery (performed: *HR*, 0.43; 95%CI: 0.42–0.44), radiotherapy (Yes: *HR*, 0.74; 95%CI: 0.72–0.76), chemotherapy (yes: *HR*, 0.56; 95%CI: 0.55–0.58), and marital status (never married: *HR*,1.41; 95%CI: 1.37–1.45; divorced or separated: *HR*, 1.33; 95%CI: 1.28–1.37; widowed: *HR*, 1.32; 95%CI: 1.32–1.43;).

**Table 3:** Multivariate Cox analyses of prognostic factors of UDTT.

**Variables**	**OS- Hazard Ratio**	**95% CI**	***P***	**CSS- Hazard Ratio**	**95% CI**	***P***
Sex			<0.001			<0.001
Female	1.00	Reference		1.00	Reference	
Male	1.08	1.06–1.10		1.05	1.02–1.07	
Age			<0.001			<0.001
<45	1.00	Reference		1.00	Reference	
45–60	1.32	1.26–1.39		1.11	1.06–1.18	
61–75	1.74	1.66–1.82		1.14	1.08–1.21	
>75	2.72	2.58–2.85		1.40	1.32–1.48	
Race			<0.001			<0.001
White	1.00	Reference		1.00	Reference	
Black	1.35	1.32–1.39		1.36	1.31–1.41	
Other	0.97	0.94–1.01		1.10	1.05–1.15	
Site			<0.001			<0.001
Oral carvity	1.00	Reference		1.00	Reference	
Pharynx	0.82	0.80–0.84		0.78	0.76–0.81	
Esophagus	3.00	2.92–3.08		3.91	3.77–4.04	
Pathological type			<0.001			<0.001
SCC	1.00	Reference		1.00	Reference	
NSCC	0.97	0.94–0.99		1.10	1.06–1.13	
Grade			<0.001			<0.001
I	1.00	Reference		1.00	Reference	
II	1.18	1.14–1.21		1.20	1.15–1.26	
III	1.18	1.14–1.22		1.24	1.19–1.30	
IV	1.08	1.02–1.15		1.13	1.05–1.23	
Stage			<0.001			<0.001
I	1.00	Reference		1.00	Reference	
II	1.58	1.53–1.63		1.97	1.87–2.06	
III	2.25	2.18–2.32		3.26	3.11–3.41	
IV	3.25	3.16–3.35		5.35	5.13–5.59	
Surgery			<0.001			<0.001
Not performed	1.00	Reference		1.00	Reference	
Performed	0.45	0.44–0.46		0.43	0.42–0.44	
Radiotherapy			<0.001			<0.001
No/Unknown	1.00	Reference		1.00	Reference	
Yes	0.66	0.65–0.67		0.74	0.72–0.76	
Chemotherapy			< 0.001			0.48
No/Unknown	1.00	Reference		1.00	Reference	
Yes	0.57	0.56–0.59		0.56	0.55–0.58	
Marital status			<0.001			<0.001
Married	1.00	Reference		1.00	Reference	
Never married	1.36	1.33–1.39		1.41	1.37–1.45	
Divorced/Sepratated	1.35	1.32–1.39		1.33	1.28–1.37	
Widowed	1.38	1.34–1.42		1.37	1.32–1.43	

^*^Including other (American Indian/AK Native, Asian/Pacific Islander) and unknowns.

### Stratified Analysis of Marital Status

**Figure 4:**
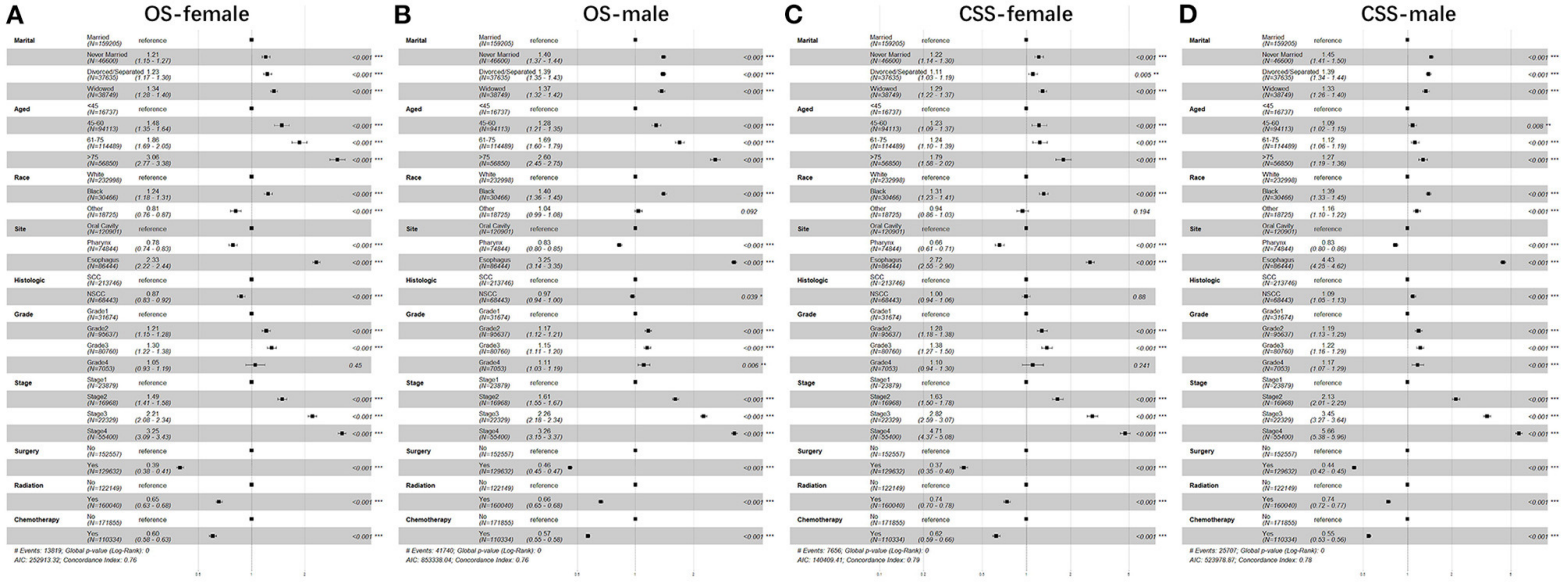
The forest plot of HR (95% CI) for OS and CSS of the patients with UDTT in each gender. HR, hazard ratio; CI confidence interval; **(A)** OS of female; **(B)** OS of male; **(C)** CSS of female; **(D)** CSS of male.

**Figure 5:**
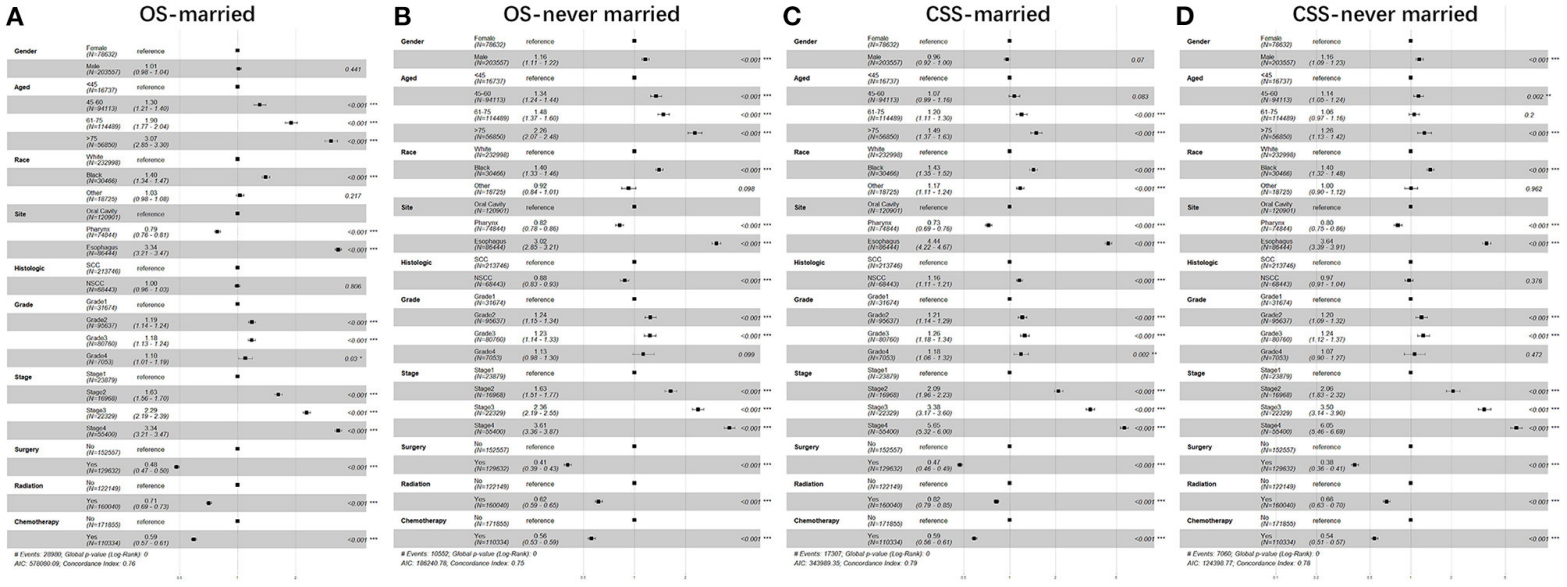
The forest plot of HR (95% CI) for OS and CSS of the patients with UDTT in subgroups of married and never married. HR, hazard ratio; CI confidence interval; **(A)** OS of married; **(B)** OS of never married; **(C)** CSS of married; **(D)** CSS of never married.

Furthermore, we studied the effect of sex, age, race, anatomic site, pathological type, grade, stage, surgery, radiotherapy, chemotherapy, and marital status on OS and CSS of patients with UDTTs by COX proportional hazard regression model (Figure 4). Men had poor OS and CSS than women in never married and divorced or separated by comparing the HR in OS (never married: *HR*, 1.40; 95%CI: 1.37–1.44; divorced or separated: *HR*, 1.39; 95%CI: 1.35–1.43) and CSS (never married: *HR*, 1.45; 95%CI: 1.41–1.50; divorced/separated: *HR*, 1.39; 95%CI: 1.34–1.44). Compared with marital status, older women were more risk in never married (Figure 4). So, we made subgroup analysis according to married and never married (Figure 5). Regarding the OS patients with UDTTs, men in the married group showed better OS than those in the never-married group (*HR*, 1.16; 95%CI: 1.11–1.22). Regarding the CSS patients with UDTTs, men in the married group showed better CSS than those in the never-married group (*HR*, 0.96; 95%CI: 0.92–1.23). Notably, compared with the unmarried group, whether for OS or CSS, age is a greater risk factor for the married group. These data showed married male patients owed better survival outcome.

## Discussion

As far as we are concerned, few researches have focused on the heterogeneity of patients with UDTT in different marital statuses with stratified comparisons. UDTT, as a type of tumor with a higher incidence and a poor prognosis (24), is closely related to psychological and behavioral factors (14, 15), and the marital relationship has a significant impact on it through psychological and behavioral differences (20, 21). Our study indicated that married patients had better OS/CSS rate of UDTT, while widowed patients were had worse OS/CSS rate. This similar survival difference was also observed in each gender, stage, and treatments.

In our study, the never-married group had gradually increased from 1975 to 2016, which is consistent with the declining marriage rate in the United States (25). At the same time, the proportion of male patients was significantly higher than that of female patients, which may be due to the fact that UDTT is related to smoking, drinking, and other living habits. The 3-year and 5-year survival rates gradually improved as the time of diagnosis approached, which indicates that with the advancement of medicine, both diagnosis and treatment of UDTT have made significant progress. Both 3-year and 5-year OS and CSS were significantly lower for men than for women. The 3-year and 5-year survival rates for Blacks were also significantly lower than Whites and other races. For the 3-year and 5-year survival rates, being married is an obvious protective factor.

Most studies suggested that unmarried patients have worse survival rates due to delayed diagnosis and under-treatment (3, 7, 26, 27). Hinyard et al. found that the probability of late-stage diagnosis among unmarried female patients was 1.18-folds higher than married female patients (28). Hershman et al. stated that unmarried subjects tended to postpone the start of adjuvant chemotherapeutic treatment after receiving the surgeries of breast carcinoma, which led to higher mortality (29). Our study found something similarly in Cox multivariate regression analysis, and we found that never-married and divorced patients in OS and CSS had greater risks for men than women, and increased age was riskier for women. From the site of UDTT, the prognosis of esophageal cancer in men was worse than that in women. Women can benefit more from the surgery. In the further stratified comparison of married and never-married status, it was found that men can benefit more from marriage than women, thus avoiding premature death caused by UDTT. The never-married group received more benefits in treatment than the married group.

There are several studies that have emphasized the importance of marriage to the patients with cancer. On the one hand, it is analyzed by psychological differences. Unmarried patients more likely display greater stress and depression when diagnosed with cancer, which can change immune function and cause tumor progression (30, 31). Meanwhile, unmarried patients lack the support and care from their spouses, so they often suffer from distressed psychological state and indulge in bad habits, just like smoking and excessive drinking, which lead to an exacerbation of tumor and poor treatment outcomes (32–34). Widowhood is a serious emotion stress, which means that social support and material support are reduced that could lead the patients to pay less attention to health and causing more non-definitive treatment even when symptoms are present (27). On the other hand, the lack of marriage has a corresponding effect on the hormones and mediators of the patient's body. The lack of social support and chronic stress may promote the secretion of cortisol (35, 36). Other studies have shown that increased psychological stress by pass through the hypothalamic-pituitary-adrenal axis to decrease the immune response and promote development of tumor. Furthermore, the release of glucocorticoids and catecholamines is regulated, which further directly influences the tumor microenvironment (37, 38), and has been implicated in cancer survival (39, 40). In contrast, it has been suggested that mates tend to encourage screening and compliance to treatment and therefore could improve treatment outcomes (4, 7).

Our study was based on a big database and involved a huge population and could give light on the impact of marital status on the prognosis in patients with UDTT. However, there are still some limitations. First, the SEER database only records marital status of patient at the time of diagnosis, not dynamically, so changes in marital status as the tumor progresses may affect the outcome of a different marital status on survival outcomes. Second, SEER database lacks corresponding records of marital quality, because disharmony and depression in marriage may negatively affect the prognosis (41). Third, a considerable proportion of patients may not have a legal marriage, but live in de facto same or opposite sex or partnerships (4). It was not until June 2015, the United States officially legalized same-sex marriage (42), and the SEER does not record it, so it is impossible to know whether there is a difference in the prognosis of tumors between same-sex and heterosexual marriages. Finally, SEER also has limited the information on adverse habits such as smoking and drinking.

In summary, our study has revealed that marital status is an independent prognostic factor of OS and CSS rates in patients with UDTTs. Compared with other marital status groups, married patients gained significantly better outcome, irrespective of different variables we studied. The never-married group performed significantly worse in OS and CSS, while men with UDTTs benefited more from marriage than women. Men with UDTTs who were never married had at higher risk than women. Thus, married status plays a significant role as a protective factor in patients with UDTTs, especially for men. Therefore, the marital status of the patients is recommended for predicting the prognosis as a clinical routine during the treatment of UDTTs, and never-married men with UDTTs also need more attention.

## Data Availability Statement

The original contributions presented in the study are included in the article/supplementary material, further inquiries can be directed to the corresponding authors.

## Ethical Statement

The SEER belongs to public databases. The patients involved in the database have obtained ethical approval. Users can download relevant data for free for research and publish relevant articles. Our study is based on open-source data, so there are no ethical issues and other conflict of interests.

## Author Contributions

MFQ as the first author and corresponding author conceived the study, wrote the initial draft, and edited the final version. JKP prepared the experimental resources and the software. QHS and HX performed data analysis and interpretation, and contributed to charts. QMC as a corresponding author assisted in manuscript revision. All authors contributed significantly to the initiation and design of the study and manuscript approval.

## Funding

This study was supported by the National Natural Science Foundation of China (No. 81771086).

## Conflict of Interest

The authors declare that the research was conducted in the absence of any commercial or financial relationships that could be construed as a potential conflict of interest.

## Publisher's Note

All claims expressed in this article are solely those of the authors and do not necessarily represent those of their affiliated organizations, or those of the publisher, the editors and the reviewers. Any product that may be evaluated in this article, or claim that may be made by its manufacturer, is not guaranteed or endorsed by the publisher.
